# Dark Triad and Interpersonal Forgiveness: The Mediating Role of Interpersonal Relationship Satisfaction

**DOI:** 10.3390/bs15020237

**Published:** 2025-02-19

**Authors:** Yaoguo Geng, Ziyang Cheng, Liping Shi, Tingting Zhan, Zhixia Hu, Wenjing Jin

**Affiliations:** 1 School of Marxism, Zhengzhou University, Zhengzhou 450001, China; 2 School of Physical Education (Main Campus), Zhengzhou University, Zhengzhou 450001, China; 3 Department of Psychology, School of Social Development and Public Policy, Fudan University, Shanghai 200433, China

**Keywords:** Dark Triad, interpersonal forgiveness, interpersonal relationship

## Abstract

The social-emotional functions associated with the Dark Triad have been widely examined. However, further research is needed to explore the nuanced relationship between the Dark Triad and interpersonal relationships, particularly in diverse cultural contexts. This study aimed to investigate the relationship between the Dark Triad and interpersonal relationship satisfaction and interpersonal forgiveness by testing 577 students with the Dirty Dozen (DD), Interpersonal Relationship Satisfaction Questionnaire (IRSQ), and the Transgression-Related Interpersonal Motivations Scale-12-Item Form (TRIM-12). Results showed that (a) overall, the Dark Triad correlated negatively with lower interpersonal relationship satisfaction and interpersonal forgiveness. (b) Narcissism differs in the pattern of correlations from Machiavellianism and psychopathy. Machiavellianism and psychopathy were positively correlated with revenge, avoidance, and interpersonal dissatisfaction and negatively correlated with interpersonal satisfaction. In contrast, narcissism was positively correlated with revenge, avoidance, interpersonal dissatisfaction, and interpersonal satisfaction. (c) The mediating models showed that individuals with higher Dark Triad scores exhibited lower interpersonal relationship satisfaction and higher levels of revenge. Individuals with higher Machiavellianism and psychopathy showed greater interpersonal relationship dissatisfaction and more pronounced avoidance. These findings highlight the nuanced role of the Dark Triad in shaping interpersonal outcomes and suggest that targeted interventions focusing on relationship satisfaction and interpersonal forgiveness could mitigate the negative social behaviors associated with these traits.

## 1. Introduction

The Dark Triad refers to a term that was introduced by Paulhus and Williams (2002) to describe a combination of personality traits characterized by specific negative features, encompassing three overlapping yet distinct personality traits (namely, Machiavellianism, psychopathy, and narcissism), which are socially undesirable and contain malevolent attributes (Rico-Bordera et al., 2025). Individuals with high levels of the Dark Triad may get ahead in self- and short-benefits (e.g., success in positions of power or short-term mating: Carter et al., 2014; Heym et al., 2019; Rauthmann & Kolar, 2013b). An in-depth study of the Dark Triad is instrumental in understanding an individual’s social behavior, mental health issues, and negative phenomena in interpersonal interactions. Research found that these traits are associated with various negative behaviors and psychological states (Li et al., 2024; Brewer et al., 2024). They may go at the expense of psychological well-being, communal welfare, and getting along (Martin et al., 2012; Rauthmann, 2012), especially for long-run interactions (Wilson et al., 1996). In recent years, due to its prominence in general populations and its important effect on social-emotional functions, the Dark Triad has gained considerable attention (Martin et al., 2012; McHoskey, 2001).

The Dark Triad has a profound impact on an individual’s cognitive, emotional, and behavioral patterns, leading to unique interaction styles in interpersonal communications. Interpersonal forgiveness, as a key factor in maintaining good interpersonal relationships, plays a crucial role in repairing damaged relationships and mitigating the harm caused by conflicts. Especially in the context of Chinese culture, forgiveness is a quality with a profound historical heritage and moral significance (as the Chinese old saying goes: the benevolent is invincible). It is not only a manifestation of personal morality but also an important principle for handling interpersonal relationships. Through forgiveness, individuals can promote harmony and stability in interpersonal relationships. Therefore, it is crucial to study the relationship between the Dark Triad and interpersonal forgiveness.

Interpersonal forgiveness is conceptualized as an emotion-focused coping strategy for dealing with interpersonal and social stress (Worthington & Scherer, 2004) and was found to correlate with a few positive outcomes such as lower levels of neuroticism and higher levels of agreeableness (Maltby et al., 2008). Conversely, those who fail to forgive are vengeful or avoidant of the transgressor and elicit desires to take revenge. Recently, a significant study was conducted to explore the relationship between revenge and the Dark Triad (Geher et al., 2021; Rasmussen & Boon, 2014). Results indicated that the likelihood of engaging in revenge against a romantic partner is correlated with the Dark Triad positively (0.38 for Machiavellianism, 0.33 for psychopathy, 0.19 for narcissism, respectively). Most notable in this study is that Machiavellianism and psychopathy may make contributions to revenge behavior indirectly through impelling factors (i.e., power/justice goals and perceptions of revenge effectiveness) rather than through inhibiting factors (i.e., interpersonal relationship goals and perceptions of revenge costliness). Another study explored the relationships between narcissism and lack of forgiveness as well as the mechanisms through which narcissism and lack of forgiveness occur (Fatfouta et al., 2015). Results showed that the relationships between narcissism and lack of forgiveness were mediated by indignation, rumination, and empathy, and empathy functioned as an inhibiting factor to suppress revenge and avoidance behavior. A recent study has also found that indignation mediated the relationship between the Dark Triad and unforgiveness (Rebrov et al., 2022). Nedeljković and other scholars have studied the relationship between the Dark Triad and forgiveness and further explored the mediating role of anger rumination (Nedeljković et al., 2023). Therefore, we hypothesize that individuals with the Dark Triad (Machiavellianism, psychopathy, and narcissism) are more likely to lack interpersonal forgiveness and tend to exhibit higher levels of revenge and avoidance.

Machiavellianism is characterized by cynical, misanthropic, and pragmatic beliefs, coldness, deception, manipulation, lack of sincerity and ethical concern, and the tendency to use interpersonal relationships as tools for personal gain, excelling in the manipulation and exploitation of others (Ali & Chamorro-Premuzic, 2010; Brewer et al., 2024; Christie & Geis, 2013; Rauthmann, 2011; Sherry et al., 2006). This behavior undermines trust, leaving others feeling deceived and exploited, making it difficult to establish genuine relationships. Psychopathy is characterized by thrill-seeking tendencies, callousness, a lack of remorse, poor impulse control, manipulative behaviors, and anti-social behaviors (Hodson et al., 2009; Rauthmann, 2011; Rico-Bordera et al., 2025). In relationships, this may manifest as disregard for others’ feelings, harmful actions without remorse (e.g., casually disclosing others’ privacy), and inflicting psychological trauma. Narcissism represents an exaggeration of self-worth and importance, superiority over others, bragging, attention- and admiration-seeking, and manipulation (Hodson et al., 2009; Raskin & Terry, 1988). Over time, this behavior can make others feel ignored, thereby triggering their dissatisfaction and displeasure, leading to tense interpersonal relationships. The Dark Triad’s demeanor in social situations is characterized by the tendency to antagonistic/exploitative behavioral style (Jones & Paulhus, 2010), which reflects their anti-sociality. Jones and Paulhus (2010) proposed that Machiavellianism, psychopathy, and narcissism all inhabit Quadrant 2 of the interpersonal circumplex space (i.e., high-agency, low-communion). This means that individuals high in the Dark Triad have negative views on people and the world and behave in an arrogant, calculating, callous, and manipulative fashion (Rauthmann & Kolar, 2013a). They manifest in aversive or problematic interpersonal outcomes (Maples-Keller & Miller, 2018). Individuals with the Dark Triad often struggle to establish and maintain healthy, positive relationships. Their behaviors and attitudes may cause distress and harm to others, thereby undermining the harmony of interpersonal relationships. Research found that interpersonal relationships and forgiveness are closely related (Jiang & Tan, 2016). Therefore, this study explored the mediating role of interpersonal relationship satisfaction between the Dark Triad and interpersonal forgiveness.

Given a collectivist culture and the importance of maintaining interpersonal harmony, it is reasonable to expect that interpersonal relationship goals may play a certain effect as an inhibiting factor (i.e., mediating factor) on unforgiveness in China. Thus, the main goal of this study was to examine the possible inhibiting effect of interpersonal relationship factors (interpersonal relationship satisfaction) on unforgiveness by testing our hypothesized model to gain a deeper understanding of the links among the Dark Triad, interpersonal relationships, and interpersonal forgiveness.

In summary, based on previous findings (mentioned above), we predicted that (a) the Dark Triad (Machiavellianism, psychopathy, and narcissism) would be correlated with low interpersonal relationship satisfaction and (b) interpersonal relationship factors play a certain mediating effect between the Dark Triad and interpersonal forgiveness. Individuals with a higher Dark Triad (Machiavellianism, psychopathy, and narcissism) may exhibit lower interpersonal relationship satisfaction and higher levels of revenge and avoidance.

## 2. Methods

### 2.1. Participants and Procedure

Participants were recruited from two universities in Zhengzhou. Using convenience sampling methods, 641 undergraduates were recruited to take part in this study in exchange for extra course credit in their psychology class. Of the initial 641 students, 64 (10%) were excluded because of incomplete data. The final sample consisted of 577 undergraduates from 20 to 24 years of age (*M* = 21.14, *SD* = 1.07), including 252 males (43.7%) and 325 females (56.3%). There was no significant difference between the males and females in age, and there was no significant difference in the demographics between the students who were included in the study and those who were excluded. The mean level of acquaintance among participants was prolonged, usually more than a year.

All procedures performed in studies involving human participants were in accordance with the ethical standards of the institutional review board at Zhengzhou University and with the 1964 Helsinki Declaration and its later amendments or comparable ethical standards. After the participants’ consents were obtained, the students completed the survey during regular class hours. Students were allowed to clarify the meaning of some questions, but their responses to the items were not influenced by the four trained researchers. Also, they were thanked and assured that their answers were completely confidential.

### 2.2. Measures

#### 2.2.1. Dirty Dozen (DD)

The Dirty Dozen is a 12-item, self-rating, and well-validated measure (Jonason & Webster, 2010) that assesses the Dark Triad traits. Participants were asked how much they agreed (1 = disagree strongly; 7 = agree strongly) with statements such as, “I have used deceit or lied to get my way” (i.e., Machiavellianism), “I tend to lack remorse” (i.e., psychopathy), and “I tend to want others to admire me” (i.e., narcissism). Items were summed together to create a total score of Machiavellianism, psychopathy, and narcissism. The Chinese version of the Dirty Dozen was documented elsewhere (Geng et al., 2015). In this study, Cronbach’s α was 0.90 for Machiavellianism, 0.76 for psychopathy, and 0.85 for narcissism.

#### 2.2.2. Interpersonal Relationship Satisfaction Questionnaire (IRSQ)

IRSQ is a 39-item, self-rating, and well-validated measure (Zhao & Zuo, 2008) that assesses interpersonal relationship satisfaction on two scales: 25 items for interpersonal satisfaction (IS, involves mutual benefit and support, similarity and compatibility, and moral quality) and 14 items for interpersonal dissatisfaction (IDS, involves communication difficulties, habit differences, and conflicts). Each item is rated on a 6-point scale anchored by 6: agree very much and 1: disagree very much. Items were summed together to create a total score of interpersonal satisfaction (range 25–150) and interpersonal dissatisfaction (range 14–84). A higher score of IS is indicative of higher levels of interpersonal satisfaction, and a higher score of IDS is indicative of higher levels of interpersonal dissatisfaction. In this study, Cronbach’s α was 0.89 for IS and 0.80 for IDS.

#### 2.2.3. Transgression-Related Interpersonal Motivation Scale 12-Item Form (TRIM-12)

TRIM-12 is a 12-item, self-rating, and well-validated measure (McCullough et al., 1998) that assesses interpersonal motivations on two scales: five items for motivation to seek revenge (REV) against the person who caused harm, and seven items for motivation to avoid (AVO) personal and psychological contact with the offender. Each item is rated on a 5-point scale anchored by 5: agree very much and 1: disagree very much. Items were summed together to create a total score of revenge (range 5–25) and avoidance motivation (range 7–35). Higher scores of REV or AVO are indicative of higher levels of revenge or avoidance motivation. TRIM-12 views interpersonal forgiveness as low motivation to seek revenge against or avoid someone who has harmed the respondent. The Chinese version of TRIM-12 was documented elsewhere (Chen et al., 2006). In this study, Cronbach’s α was 0.85 for revenge and 0.83 for avoidance.

## 3. Results

### 3.1. Sex Differences

Table 1 shows the means and standard deviations for all variables. As expected, males reported higher levels of Machiavellianism, psychopathy, and interpersonal dissatisfaction than females. Females reported higher levels of interpersonal satisfaction than males. Females reported lower levels of revenge motivation than males.

### 3.2. Correlations

As shown in Table 2, correlations among the Dark Triad are almost positive and significant, except for the relationship between psychopathy and narcissism (*r* = 0.051, *p* > 0.05). Also, a similar pattern of correlations was found for Machiavellianism and psychopathy, which were both positively associated with revenge, avoidance, and interpersonal dissatisfaction (*r* between 0.153 and 0.475, *p* < 0.01) but negatively associated with interpersonal satisfaction (*r* = −0.253 and −0.267, respectively, *p* < 0.01). As for narcissism, a different pattern of correlations was obtained: this trait was positively associated with revenge (*r* = 0.206, *p* < 0.01), avoidance (*r* = 0.208, *p* < 0.01), and interpersonal dissatisfaction (*r* = 0.082, *p* < 0.05) and also displayed a positive correlation with interpersonal satisfaction (*r* = 0.115, *p* < 0.05). These results indicate that narcissism differs in the pattern of correlations from Machiavellianism and psychopathy. In addition, revenge and avoidance were associated with dissatisfaction with interpersonal relationships.

### 3.3. Structural Equation Modeling (SEM)

We tested this model using the AMOS 24.0 statistical program and maximum likelihood estimation. We also calculated indirect path coefficients showing relationships between factors mediated by intervening variables in the model. In the current study, the model fit was evaluated with the following indicators: *X*^2^/*df* < 5; RMSEA < 0.08, GFI, AGFI, CFI, and NFI > 0.95.

**Figure 1:**
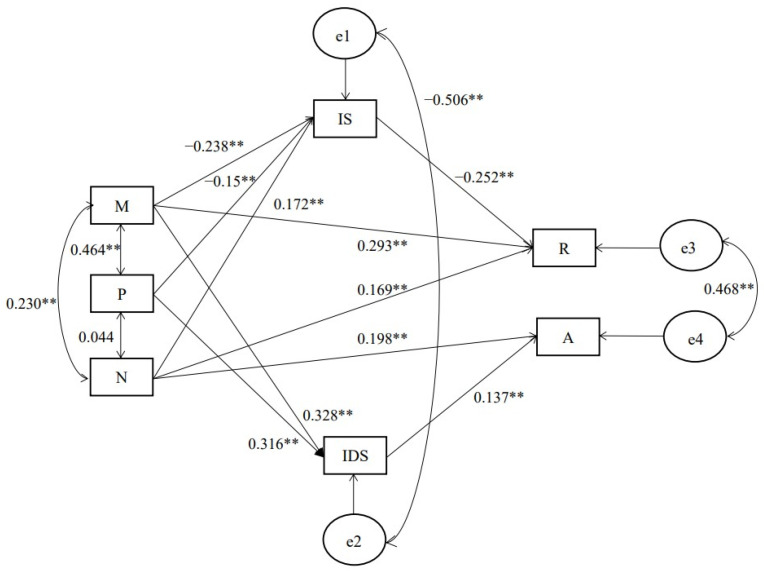
Hypothesized model testing the relationship between Dark Triad and interpersonal forgiveness. Note: M = Machiavellianism; P = psychopathy; N = narcissism; R = revenge; A = avoidance; IS = interpersonal satisfaction; IDS = interpersonal dissatisfaction; ** *p* < 0.01.

The hypothesized model included two mediators, namely, interpersonal satisfaction and interpersonal dissatisfaction. In line with predictions, the Dark Triad traits were hypothesized either to make direct contributions to interpersonal forgiveness or to affect interpersonal forgiveness through interpersonal satisfaction and interpersonal dissatisfaction. The original model did not fit the data well (*X*^2^ = 6.55, *df* = 1, *X*^2^/*df* = 6.55, RMSEA = 0.098, GFI = 0.883, AGFI = 0.825, NFI = 0.881, CFI = 0.912), indicating some adjustment was necessary. In line with modification indices, five theoretically relevant paths were removed, namely, from Machiavellianism to avoidance, from psychopathy to revenge, from psychopathy to avoidance, from narcissism to interpersonal dissatisfaction, and from interpersonal dissatisfaction to revenge. Results indicated that the modified model (Figure 1) explained the data very well (*X*^2^ = 11.92, *df* = 6, *X*^2^/*df* = 1.99, RMSEA = 0.025, GFI = 0.994, AGFI = 0.973, NFI = 0.988, CFI = 0.994).

As shown in Figure 1, the greater their scores on Machiavellianism and narcissism, the more undergraduates endorsed revenge behavior; the greater their scores on narcissism, the more undergraduates endorsed avoidance behavior; the more undergraduates endorsed interpersonal satisfaction, the less they endorsed revenge behavior; the more undergraduates endorsed interpersonal dissatisfaction, the more they endorsed avoidance behavior.

Additionally, as indicated by the indirect path coefficients relating the Dark Triad to revenge and avoidance, Machiavellianism, psychopathy, and narcissism were indirectly correlated with revenge through interpersonal satisfaction and Machiavellianism and psychopathy were indirectly correlated with avoidance through interpersonal dissatisfaction. These results suggest that interpersonal relationship satisfaction may have a certain effect as an inhibiting factor in revenge-taking.

Specifically, the relationship between Machiavellianism and revenge is partially mediated by interpersonal satisfaction (a × b 95% CI 0.279, 0.415), the relationship between Machiavellianism and avoidance is fully mediated by interpersonal dissatisfaction (a × b 95% CI −0.006, 0.232), the relationship between psychopathy and revenge is fully mediated by interpersonal satisfaction (a × b 95% CI 0.095, 0.274), the relationship between psychopathy and avoidance is also fully mediated by interpersonal dissatisfaction (a × b 95% CI −0.015, 0.243), and the relationship between narcissism and revenge is partially mediated by interpersonal satisfaction (a × b 95% CI 0.155, 0.294).

## 4. Discussion and Conclusions

In the present study, males were found to score significantly higher than females on Machiavellianism and psychopathy, which is consistent with previous studies (Jonason & Tost, 2010; Jonason & Webster, 2010; Zhu et al., 2021). Males also reported higher levels of interpersonal dissatisfaction and revenge motivation than females, suggesting an antagonistic behavioral style. In addition, females reported higher levels of interpersonal satisfaction than males, indicating a friendly disposition.

As predicted, Machiavellianism and psychopathy were both positively associated with interpersonal dissatisfaction and negatively associated with interpersonal satisfaction. These results suggest that individuals high in Machiavellianism or psychopathy often experience diminished interpersonal relationship satisfaction.

In this study, a distinct finding for narcissism emerged when looking at the pattern of correlations among self-report Dark Triad and interpersonal relationship satisfaction. That is, in contrast with Machiavellianism and psychopathy, narcissism displayed a unique positive correlation with interpersonal satisfaction and a weak positive correlation with interpersonal dissatisfaction. More notable than these findings is the relationship between narcissism and psychopathy, striking in that Dark Triad traits are intrinsically overlapping (Jones & Paulhus, 2017; Persson, 2019). These results seem to suggest that there is a feature that distinguishes narcissism from the remaining two Dark Triad traits, Machiavellianism and psychopathy, which is in line with some studies (Rauthmann & Kolar, 2013b; Shang et al., 2022; Veselka et al., 2012; Vize et al., 2018).

There are several possible reasons for the finding that narcissism differs in the pattern of correlations from Machiavellianism and psychopathy. First, compared with Machiavellianism and psychopathy, narcissism has underlying heterogeneity among Dark Triad traits. Rauthmann and Kolar (2013b) argued that Machiavellianism and psychopathy form a “Malicious Two” because of common core elements—callousness and exploitation. Second, this finding suggests that youths endorsed more egocentric standards and narcissistic behaviors (such as charmingness, agency, leadership, and boldness) and might not be judged as more negative (Rauthmann & Kolar, 2013b). This result also reflects the fact that, similar to their occidental peers, some narcissistic behaviors may be desired by Chinese youths. Going forward, it appears economic and social development changed Chinese people’s thinking and traditional collectivist values. Third, this result may show that the Dirty Dozen presently being used to assess narcissism may not be capturing sufficiently the maladaptive features of narcissistic behaviors (Veselka et al., 2012). In other words, there are some doubts about the validity of the narcissism factor of the Dirty Dozen (Geng et al., 2015).

As hypothesized, the present study was able to provide insight into the underlying psychological systems that the Dark Triad associated with interpersonal forgiveness. Results showed that the relationship between Machiavellianism and revenge is partially mediated by interpersonal satisfaction, the relationship between Machiavellianism and avoidance is fully mediated by interpersonal dissatisfaction, the relationship between psychopathy and revenge is fully mediated by interpersonal satisfaction, the relationship between psychopathy and avoidance is also fully mediated by interpersonal dissatisfaction, and the relationship between narcissism and revenge is partially mediated by interpersonal satisfaction. In short, in the present hypothetical context and with the variables under examination, we found that (a) the Dark Triad’s predictive effect on revenge behavior was inhibited by increasing interpersonal satisfaction; (b) the predictive effect of the “Malicious Two” (i.e., Machiavellianism and psychopathy) on avoidance behavior was compelled by increasing interpersonal dissatisfaction. In other words, interpersonal relationship goals play a promoting effect on the forgiveness attitudes of the Dark Triad toward others, improving the unforgiving attitude toward others.

However, apart from indirect effects, Machiavellianism and narcissism may still make direct contributions to unforgiveness. These results mean that, regardless of their interpersonal relationship satisfaction, there are other factors that lead those high in Machiavellianism or high in narcissism that will be less forgiving, especially individuals high in Machiavellianism (Rasmussen & Boon, 2014; Rebrov et al., 2022). Individuals with high Machiavellianism, due to their deep-seated instrumental worldview and lack of empathy, suffer a significant impairment in their ability to empathize with others’ needs. This leads to their lack of forgiveness. They tend to justify their actions through “moral rationalization”, seeing deception or harm as a necessary means to achieve goals, thus reducing moral guilt. Moreover, they typically exhibit a “hostile attribution bias” when interpreting others’ intentions. Even neutral behaviors are easily misconstrued as provocative, making their lack of forgiveness seem justifiable (Međedović, 2024).

In summary, the Dark Triad is associated with lower interpersonal relationship satisfaction and reduced interpersonal forgiveness. Narcissism exhibits a distinct correlation pattern compared to Machiavellianism and psychopathy, particularly in its relationship with interpersonal satisfaction. Individuals with a higher Dark Triad tend to demonstrate lower interpersonal satisfaction and higher revenge. Furthermore, those with elevated Machiavellianism and psychopathy show greater interpersonal dissatisfaction and more pronounced avoidance behaviors.

## 5. Implication

This study holds significant theoretical and practical implications. Theoretically, it deepens the Dark Triad personality theory and reveals the unique correlation patterns of narcissism with interpersonal relationship satisfaction and interpersonal forgiveness. It expands the research scope by introducing the Dark Triad into interpersonal relationship studies. Moreover, it clarifies the relationship between the Dark Triad and interpersonal relationship, thus improving the social-emotional function framework.

Practically, it provides valuable guidance for different fields. In education and workplace management, it helps educators and managers identify individuals with a high Dark Triad and take appropriate measures to promote healthy development and enhance work efficiency. Mental health intervention and social policy-making enable professionals to formulate effective treatment plans and harmonious social policies, ultimately improving mental health and reducing social conflicts.

## 6. Limitation and Prospect

There are several limitations to the present study that should be considered. First, this study was a cross-sectional design. To elucidate the stability of the Dark Triad and more definitively test the mechanism, a longitudinal study is needed. Second, social desirability and the Middle Way (an Eastern view that refers to always solving problems in a peaceful, mutually respectful, and pragmatic manner) probably played a role in this study and made undergraduates provide biased reports on forgiveness attitudes. Third, the Dirty Dozen presently being used to assess narcissism may not accurately and completely capture sufficiently the maladaptive features of narcissistic behaviors (Veselka et al., 2012). Thus, future research should take into account these important factors and take the results reported here further by subjecting them to more rigorous testing. Finally, although it is important to study the Dark Triad in a community sample, it limits the generalizability of the results to people from clinical and forensic samples. Additionally, this study used undergraduate students as samples and adopted convenience sampling, which may lead to sample homogenization and make it difficult to generalize the conclusions to other groups. Future research should adopt multiple sampling methods and expand them to high-risk samples (or other groups) to determine whether similar relationships among the Dark Triad, interpersonal relationship satisfaction, and interpersonal forgiveness emerge.

## Figures and Tables

**Table 1:** Sex differences for all variables.

	Total (*n* = 577)	Male (*n* = 252)	Female (*n* = 325)	*t*	*p*
1 Machiavellianism	8.50 ± 4.72	9.81 ± 5.15	7.54 ± 4.06	5.92	0.000
2 Psychopathy	8.81 ± 4.21	9.79 ± 4.34	8.06 ± 3.95	4.99	0.000
3 Narcissism	18.54 ± 4.89	18.32 ± 5.15	18.77 ± 4.65	−1.09	0.275
4 Revenge	12.63 ± 4.46	13.31 ± 4.41	12.09 ± 4.44	3.29	0.001
5 Avoidance	22.21 ± 5.96	21.69 ± 6.03	22.60 ± 5.89	−1.82	0.069
6 Interpersonal satisfaction	102.27 ± 16.93	98.69 ± 17.73	105.18 ± 15.54	−4.67	0.000
7 Interpersonal dissatisfaction	30.50 ± 11.55	32.99 ± 11.66	28.55 ± 11.10	4.66	0.000

**Table 2:** Intercorrelations’ matrix.

	1	2	3	4	5	6	7
1 Machiavellianism	1						
2 Psychopathy	0.479 **	1					
3 Narcissism	0.228 **	0.051	1				
4 Revenge	0.418 **	0.241 **	0.206 **	1			
5 Avoidance	0.157 **	0.153 **	0.208 **	0.489 **	1		
6 Interpersonal satisfaction	−0.267 **	−0.253 **	0.115 *	−0.296 **	−0.019	1	
7 Interpersonal dissatisfaction	0.475 **	0.469 **	0.082 *	0.360 **	0.187 **	−0.564 **	1

Note: * *p* < 0.05, ** *p* < 0.01.

## Data Availability

The data that support the findings of this study are available on request from the corresponding author. The data are not publicly available due to privacy or ethical restrictions. The data supporting the conclusions of this article cannot be posted in a public repository because participants did not consent to such data sharing in the initial consent form. The anonymized data will be made available by the authors, without undue reservation, to any qualified researcher.
